# Prognostic and predictive value of tumor deposits in advanced signet ring cell colorectal cancer: SEER database analysis and multicenter validation

**DOI:** 10.1186/s12957-024-03362-0

**Published:** 2024-04-22

**Authors:** Fuchao Li, Lei Liu, Qingzhao Feng, Xiaohong Wang, Fang Liu, Li Yang, Lin Miao, Weiming Wang, Guozhong Ji, Chenggong Yu

**Affiliations:** 1 Department of Gastroenterology, Nanjing Drum Tower Hospital Clinical College of Nanjing Medical University, Nanjing, 210008 China; 2 Department of Geriatrics, Nanjing Drum Tower Hospital, The Affiliated Hospital of Nanjing University Medical School, Nanjing, 210000 China; 3 Medical Centre for Digestive Diseases, The Second Affiliated Hospital of Nanjing Medical University, 121 Jiangjiayuan Road, Nanjing, 210046 China; 4 Department of Gastroenterology, The Affiliated Yixing Hospital of Jiangsu University, Yixing, Jiangsu 214200 China; 5 Department of General Surgery, Nanjing Drum Tower Hospital Clinical College of Nanjing Medical University, Nanjing, 210008 China; 6 Department of Gastroenterology, Xuzhou Central Hospital, Xuzhou, Jiangsu Province 221009 China; 7 Department of Oncology, Yixing Hospital Affiliated to Medical College of Yangzhou University, Yixing, Jiangsu Province 214200 China

**Keywords:** Colorectal SRCC, Tumor deposits, Risk factor, Predictive model, Nomogram

## Abstract

**Background:**

Colorectal signet-ring cell carcinoma (SRCC) is a rare cancer with a bleak prognosis. The relationship between its clinicopathological features and survival remains incompletely elucidated. Tumor deposits (TD) have been utilized to guide the N staging in the 8th edition of American Joint Committee on Cancer (AJCC) staging manual, but their prognostic significance remains to be established in colorectal SRCC.

**Patients and methods:**

The subjects of this study were patients with stage III/IV colorectal SRCC who underwent surgical treatment. The research comprised two cohorts: a training cohort and a validation cohort. The training cohort consisted of 631 qualified patients from the SEER database, while the validation cohort included 135 eligible patients from four independent hospitals in China. The study assessed the impact of TD on Cancer-Specific Survival (CSS) and Overall Survival (OS) using Kaplan-Meier survival curves and Cox regression models. Additionally, a prognostic nomogram model was constructed for further evaluation.

**Results:**

In both cohorts, TD-positive patients were typically in the stage IV and exhibited the presence of perineural invasion (PNI) (*P* < 0.05). Compared to the TD-negative group, the TD-positive group showed significantly poorer CSS (the training cohort: HR, 1.87; 95% CI, 1.52–2.31; the validation cohort: HR, 2.43; 95% CI, 1.55–3.81; all *P* values < 0.001). This association was significant in stage III but not in stage IV. In the multivariate model, after adjusting for covariates, TD maintained an independent prognostic value (*P* < 0.05). A nomogram model including TD, N stage, T stage, TNM stage, CEA, and chemotherapy was constructed. Through internal and external validation, the model demonstrated good calibration and accuracy. Further survival curve analysis based on individual scores from the model showed good discrimination.

**Conclusion:**

TD positivity is an independent factor of poor prognosis in colorectal SRCC patients, and it is more effective to predict the prognosis of colorectal SRCC by building a model with TD and other clinically related variables.

**Supplementary Information:**

The online version contains supplementary material available at 10.1186/s12957-024-03362-0.

## Introduction

Colorectal cancer (CRC) is one of the most common cancers in the world, and the incidence and mortality of CRC are gradually increasing in developing countries [1]. In recent years, the survival of patients with CRC has gradually improved with the continuous update of examination and treatment methods. Colorectal signet-ring cell carcinoma (SRCC) is a rare tumor type, accounting for approximately 1% of all CRC [2, 3]. Histologically, colorectal SRCC is characterized by the presence of nuclei that are crescent-shaped and resemble rings, hence the name, which are off-centered in over 50% of tumor cells [3–5]. However, in recent years, the incidence of colorectal SRCC has been rising, and it is typically diagnosed at advanced stages with high rates of lymph node and peritoneal metastases [6]. Consequently, its 5-year survival rate is only about 20%, which poses a significant public health challenge [2, 7]. Most studies on colorectal SRCC are case reports or small-sample retrospective studies due to its rarity.

Tumor deposits (TD) is a common pathological detection marker, observed in 20% of CRC [8]. Some studies considered TD as isolated positive lymph nodes [9–11]. In the 5th edition of American Joint Committee on Cancer (AJCC) TNM staging system, TD was introduced into the guideline for the first time, and it was clear that lymph nodes smaller than 3 mm were classified as TD, and those larger than 3 mm were classified as lymph nodes [12]. In the 7th and 8th editions of the AJCC TNM staging system, TD was defined as cancerous nodules without histological aspects of lymph nodes, vessels, and peripheral nerve infiltration, irrespective of contour or size [13, 14].

TD was used to guide the N staging for CRC absent of lymph node metastasis and were often regarded as an independent indicator of poor prognosis [15]. To date, no research has investigated the existence of TD in colorectal SRCC. In this study, we aim to investigate the prognostic significance of TD in stage III/IV colorectal SRCC.

## Patients and methods

### Study design and patients

**Fig. 1:**
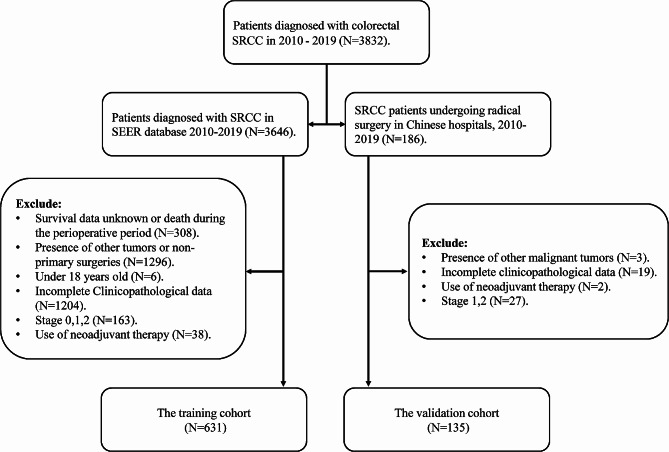
Study flowchart displaying the selection of patients with stage III/IV colorectal SRCC according to exclusion criteria. SRCC, signet-ring cell carcinoma

This study included patients diagnosed with stage III/IV colorectal SRCC who received surgical treatment. Excluded cases primarily consisted of patients lacking complete clinicopathological information, such as details on tumor subsite, size, grade, T stage, N stage, M stage, Carcinoembryonic antigen (CEA) levels, perineural invasion (PNI), TD status, and survival duration. Additionally excluded were perioperative deaths and patients outside the age range of 18 to 100 years. Two cohorts were involved in this study: a training cohort and a validation cohort. The training cohort comprised 631 eligible patients from the SEER database between 2010 and 2019 (http://seer.cancer.gov/seerstat/). The validation cohort included 135 eligible patients from four independent Chinese hospitals between 2010 and 2019. There were 36 cases in Nanjing Drum Tower Hospital, 17 cases in the Affiliated Yixing Hospital of Jiangsu University, 64 cases in Xuzhou Central Hospital and 18 cases in the Second Affiliated Hospital of Nanjing Medical University. The study flow chart is presented in Fig. 1. The ethics committees at each center approved the ethical consent of this research.

### Variables

The clinicopathological factors investigated in this study included the diagnosis date, gender, age, tumor location, size, grade, T stage, N stage, M stage, CEA, PNI, TD, chemotherapy status, and survival outcomes. CEA tests in Chinese patient data were performed by direct chemiluminescence method in four hospitals in China, and CEA level > 5ng/mL was considered positive [16]. The TNM staging was determined according to the 8th edition of the AJCC guideline. The colon was anatomically divided into the left and right sides by the splenic flexure [17]. Overall survival (OS) was measured from the diagnosis date to the death date, while cancer-specific survival (CSS) was calculated from the diagnosis date to the date of cancer-related death.

### Statistical analysis

Categorical variables were assessed using the chi-square test, while continuous variables were evaluated using the Wilcoxon rank-sum test. Survival analysis was conducted using the Kaplan-Meier method and Cox regression model. A visual nomogram model was constructed. Further internal validation was performed by bootstrap, and external validation using the Chinese dataset. The discriminative ability was evaluated using the area under the curve (AUC) and concordance index (C-index), while calibration plots were employed to assess calibrating ability. Statistical significance was considered at a *P*-value of less than 0.05 (*P* < 0.05). Statistical analysis was carried out using GraphPad Prism 9.4.1, SPSS version 21.0, and R version 3.6.1.

## Results

### Patient clinicopathologic characteristics

Patient characteristics were listed in Table 1. The median age was 65 years (range, 18–96 years) in the training cohort and 59 years (range, 16–88 years) in the validation cohort. The 3- and 5-year Cancer-Specific Survival (CSS) rates were 36.1% and 29.0% in the training cohort, and 42.2% and 31.6% in the validation cohort, respectively. The 3- and 5-year overall survival (OS) rates were 32.0% and 23.5% in the training cohort, and 39.2% and 27.2% in the validation cohort, respectively. In the training cohort, there were 314 TD-positive patients, among them, 157 (50.0%) were in stage III, 157 (50.0%) were in stage IV, and 38 (12.1%) had N1a/b,7 (2.2%) had N1c, and 269 (85.7%) had N2. Meanwhile, the characteristics of TD positive patients are ≤ 65 years old, tumor diameter greater than 5 cm, poor differentiation, T4 stage, ≥ 4 positive lymph nodes, distant metastasis, PNI and CEA positive. Meanwhile, in the validation cohort, there were 56 TD-positive patients, with 30 (53.6%) in stage III, 26 (46.4%) in stage IV, 13 (23.2%) with N1a/b, and 43 (76.8%) with N2. Patients in the validation cohort were typically in stage IV with the presence of PNI.

**Table 1:** Patient characteristics by tumor deposits in III/IV SRCC in the two cohorts

Characteristic	Training cohort			Validation cohort		
	TD-negative *N* = 317(%)	TD-positive *N* = 314(%)	*P* value	TD-negative *N* = 79(%)	TD-positive *N* = 56(%)	*P* value
Age (year)			0.002			0.385
≤ 65	140(44.2)	177(56.4)		47(59.5)	29(51.8)	
> 65	177(55.8)	137(43.6)		32(40.5)	27(48.2)	
Size			0.010			0.730
≤ 5 cm	158(49.8)	113(36.0)		42(53.2)	28(50.0)	
> 5 cm	159(50.2)	201(64.0)		37(46.8)	28(50.0)	
Gender			0.265			0.590
Male	148(46.7)	161(51.3)		48(60.8)	37(66.1)	
Female	169(53.3)	153(48.7)		31(39.2)	19(33.9)	
Subsite			0.188			0.337
Right	224(70.7)	202(64.3)		22(27.8)	13(23.3)	
Left	82(25.9)	102(32.5)		19(24.1)	20(35.7)	
Rectal	11(3.4)	10(3.3)		38(48.1)	23(42.1)	
Grade			0.038			0.087
Well	21(6.6)	9(2.9)		4(5.1)	0	
Poor	296(93.4)	305(97.1)		75(94.9)	56(100)	
T stage			< 0.001			0.159
T1-3	177(55.8)	88(28.0)		39(49.4)	20(35.7)	
T4	140(44.2)	226(72.0)		40(50.6)	36(64.3)	
N stage			< 0.001			0.836
< 4 nodes	112(35.3)	45(14.3)		17(21.5)	13(23.2)	
≥ 4 nodes	205(64.7)	269(85.7)		62(78.5)	43(76.8)	
TNM stage			< 0.001			0.005
III	249(78.5)	157(50.0)		61(77.2)	30(53.6)	
IV	68(21.5)	157(50.0)		18(22.8)	26(46.4)	
PNI			< 0.001			0.008
Absent	232(73.2)	147(46.8)		50(63.3)	22(39.3)	
Present	85(26.8)	167(53.2)		29(36.7)	34(60.7)	
CEA			0.008			0.487
Negative	153(48.3)	118(37.6)		42(53.2)	26(46.4)	
Positive	164(51.7)	196(62.4)		37(46.8)	30(53.6)	
Chemotherapy			0.074			0.455
No	112(35.3)	90(28.7)		22(27.8)	19(33.9)	
Yes	205(64.7)	224(71.3)		57(72.2)	37(66.1)	

### Prognostic value of TD by Kaplan-Meier

**Fig. 2:**
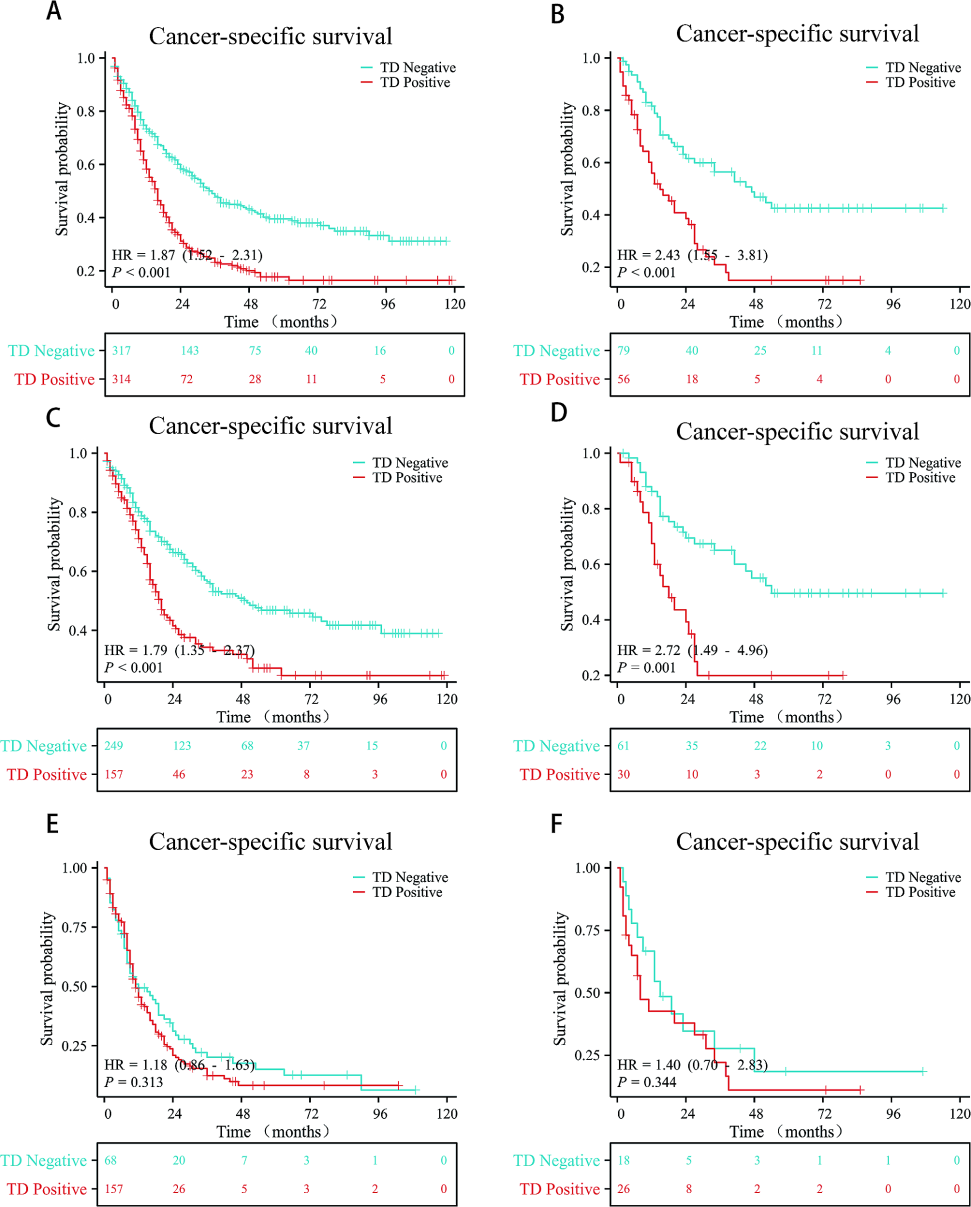
Kaplan-Meier curves of CSS for patients stratified by TD. **A** All the training cohort patients. **B** All the validation cohort patients. **C** Stage III patients in the training cohort. **D** Stage III patients in the validation cohort. **E** Stage IV patients in the training cohort. **F** Stage IV patients in the validation cohort. CSS, cancer-specific survival, TD, tumor deposits

In the training cohort, compared to the TD-negative group, the TD-positive group showed a significantly poorer CSS rate (HR, 1.87; 95% CI, 1.52–2.31; *P* < 0.001; Fig. 2A), with 5-year CSS rates of 17.7% vs. 39.5%. Similar trends were observed in the validation cohort (HR, 2.43; 95% CI, 1.55–3.81; *P* < 0.001; Fig. 2B), with 5-year CSS rates of 15.0% vs. 42.5%. Further stratified analysis revealed a significant association between TD positivity and poorer CSS rate in stage III (training cohort: HR, 1.79; 95% CI, 1.33–2.37; *P <* 0.001; Fig. 2C; validation cohort: HR, 2.72; 95% CI, 1.49–4.96; *P* = 0.001; Fig. 2D). However, in stage IV, the prognostic value of TD was not significant in both the training cohort (HR, 1.18; 95% CI, 0.86–1.63; *P* = 0.313; Fig. 2E) and the validation cohort (HR, 1.40; 95% CI, 0.70–2.83; *P* = 0.344; Fig. 2F). The results for OS were similar to those observed for CSS (Supplementary Fig. 1).

### TD and other independent risk factors analysis

Univariable analysis indicated that in the training cohort, tumor size, N stage, T stage, TNM stage, PNI, TD, CEA and chemotherapy were significantly associated with the CSS rate (*P* < 0.05). Incorporating these eight variables into a multivariate model revealed that N stage, T stage, TNM stage, TD, CEA, and chemotherapy were independent prognostic factors for CSS rate (*P* < 0.05, Table 2). Similarly, in both univariable and multivariable analysis, TD showed prognostic value for OS rate (*P* < 0.05, Table 3). In the validation cohort, after adjusting for tumor size, N stage, T stage, TNM stage, PNI, CEA, and chemotherapy variables, TD positivity retained prognostic value for CSS and OS rate (for CSS, HR: 1.75 (1.04–2.94); *P* = 0.035; for OS, 1.97 (1.21–3.18); *P* = 0.006; Table 4).

**Table 2:** Univariate and multivariate analysis of cancer-specific survival in the training cohort

Characteristic	Univariate analysis		Multivariate analysis	
	HR (95%CI)	*P* value	HR (95%CI)	*P* value
Age	1.00 (0.99–1.01)	0.186		
Size				
≤ 5 cm	Ref.		Ref.	
> 5 cm	1.43 (1.16–1.76)	0.001	1.113 (0.90–1.38)	0.333
Gender				
Male	Ref.			
Female	0.91 (0.74–1.12)	0.383		
Subsite		0.827		
Right	Ref.			
Left	0.98 (0.77–1.23)	0.867		
Rectal	0.83 (0.45–1.52)	0.543		
Grade				
Well	Ref.			
Poor	0.98 (0.62–1.55)	0.923		
T stage				
T1-3	Ref.		Ref.	
T4	1.95 (1.57–2.42)	< 0.001	1.460 (1.16–1.83)	0.001
N stage				
< 4 nodes	Ref.		Ref.	
≥ 4 nodes	2.42 (1.84–3.19)	< 0.001	2.05 (1.54–2.73)	< 0.001
TNM stage				
III	Ref.		Ref.	
IV	2.48 (2.02–3.05)	< 0.001	1.90 (1.51–2.38)	< 0.001
PNI				
Absent	Ref.		Ref.	
Present	1.40 (1.14–1.72)	0.001	1.03 (0.83–1.27)	0.817
TD				
Negative	Ref.		Ref.	
Positive	1.86 (1.51–2.28)	< 0.001	1.42 (1.13–1.78)	0.003
CEA				
Negative	Ref.		Ref.	
Positive	1.67 (1.35–2.06)	< 0.001	1.38 (1.11–1.72)	0.004
Chemotherapy				
No	Ref.		Ref.	
Yes	0.55 (0.45–0.69)	< 0.001	0.46 (0.37–0.56)	< 0.001

**Table 3:** Univariate and multivariate analysis of overall survival in the training cohort

Characteristic	Univariate analysis		Multivariate analysis	
	HR (95%CI)	*P* value	HR (95%CI)	*P *value
Age	1.01 (1.00-1.02)	0.003	1.01 (1.00-1.02)	0.003
Size				
≤ 5 cm	Ref.		Ref.	
> 5 cm	1.39 (1.15–1.69)	0.001	1.12 (0.91–1.37)	0.292
Gender				
Male	Ref.			
Female	0.94 (0.78–1.14)	0.538		
Subsite		0.729		
Right	Ref.			
Left	0.94 (0.76–1.16)	0.538		
Rectal	0.85 (0.49–1.48)	0.568		
Grade				
Well	Ref.			
Poor	1.01 (0.65–1.57)	0.957		
T stage				
T1-3	Ref.		Ref.	
T4	1.83 (1.50–2.24)	< 0.001	1.43 (1.16–1.77)	0.001
N stage				
< 4 nodes	Ref.		Ref.	
≥ 4 nodes	2.15 (1.68–2.76)	< 0.001	2.01 (1.55–2.61)	< 0.001
TNM stage				
III	Ref.		Ref.	
IV	2.22(1.83–2.70)	< 0.001	1.83(1.47–2.27)	< 0.001
PNI				
Absent	Ref.		Ref.	
Present	1.35(1.11–1.63)	0.002	1.04(0.85–1.27)	0.727
TD				
Negative	Ref.		Ref.	
Positive	1.75(1.44–2.12)	< 0.001	1.44(1.16–1.78)	< 0.001
CEA				
Negative	Ref.		Ref.	
Positive	1.61(1.33–1.97)	< 0.001	1.25(1.01–1.55)	0.039
Chemotherapy				
No	Ref.		Ref.	
Yes	0.50(0.40–0.60)	< 0.001	0.46(0.371–0.57)	< 0.001

**Table 4:** Multivariate analysis of cancer-specific survival and overall survival in the validation cohort

Characteristic	CSS	*P* value	OS	*P *value
Size				
≤ 5 cm	Ref.		Ref.	
> 5 cm	1.13(0.71–1.77)	0.615	1.15(0.74–1.78)	0.528
T stage				
T1-3	Ref.		Ref.	
T4	1.59(0.96–2.64)	0.073	1.31(0.83–2.07)	0.254
N stage				
< 4 nodes	Ref.		Ref.	
≥ 4 nodes	1.42(0.77–2.62)	0.266	1.10(0.64–1.87)	0.737
TNM stage				
III	Ref.		Ref.	
IV	1.89(1.15–3.12)	0.012	1.58(0.98–2.53)	0.061
PNI				
Absent	Ref.		Ref.	
Present	1.99(1.18–3.34)	0.010	1.84(1.13-3.00)	0.014
TD				
Negative	Ref.		Ref.	
Positive	1.75(1.04–2.94)	0.035	1.96(1.21–3.18)	0.006
CEA				
Negative	Ref.		Ref.	
Positive	2.04(1.27–3.27)	0.003	1.90(1.23–2.96)	0.004
Chemotherapy				
No	Ref.		Ref.	
Yes	1.21(0.73-2.00)	0.452	1.00(0.63–1.57)	0.981

### Nomogram construction and validation

**Fig. 3:**
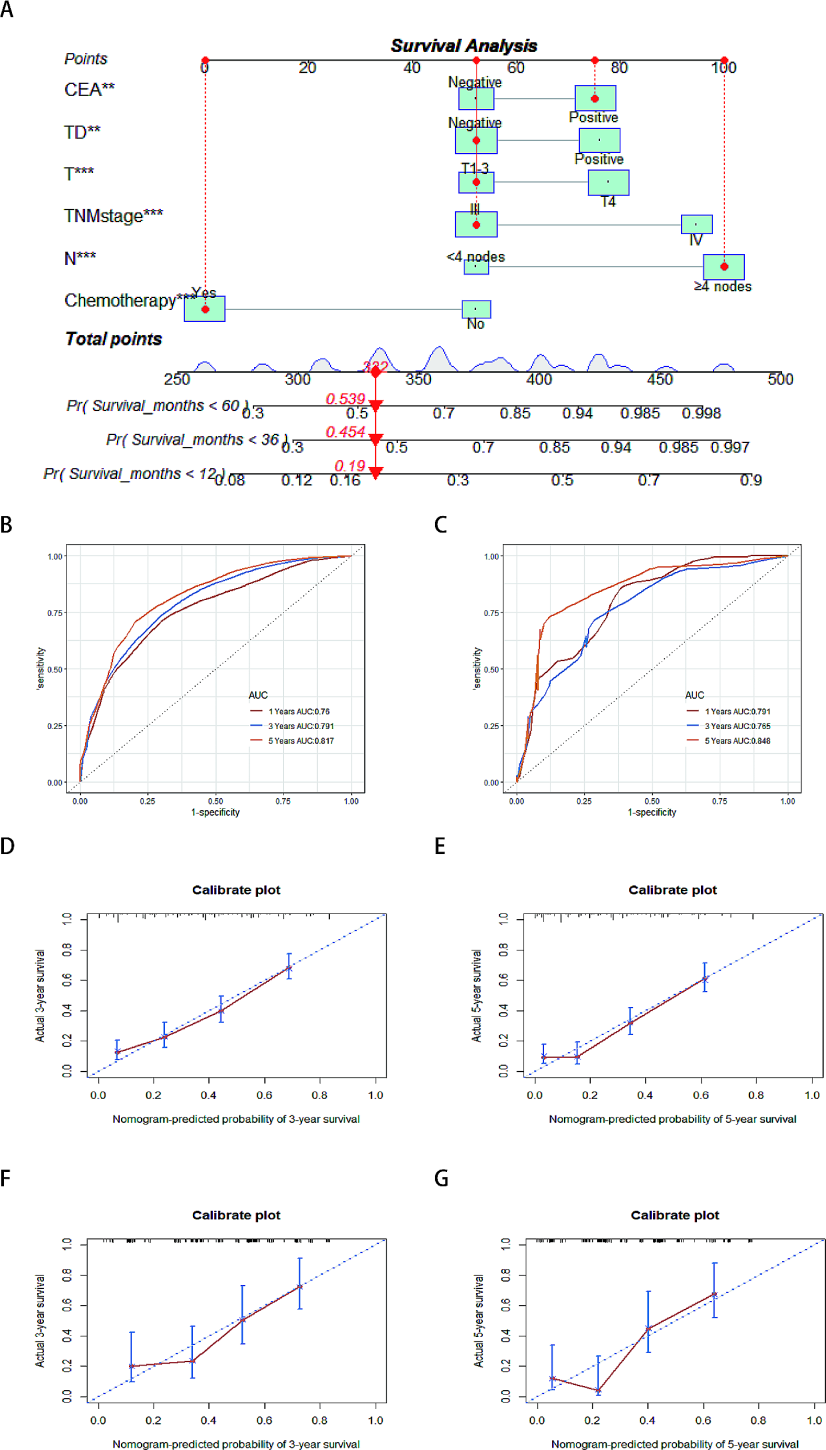
Nomogram model of training cohort and validation cohort. **A** Nomogram model predicting the 1-, 3-and 5-year CSS in patients with stage III/IV colorectal SRCC. **B** AUC comparison of CSS nomogram, 1-, 3-and 5-year AUC of CSS nomogram using training cohort; **C** Using validation cohort. The calibration curves for predicting patient CSS in the training cohort at **D** 3 year and **E** 5 years, and in the validation cohort at **F** 3 year and **G** 5 years. SRCC, signet-ring cell carcinoma, AUC, area under the curve, CSS, cancer-specific survival

**Fig. 4:**
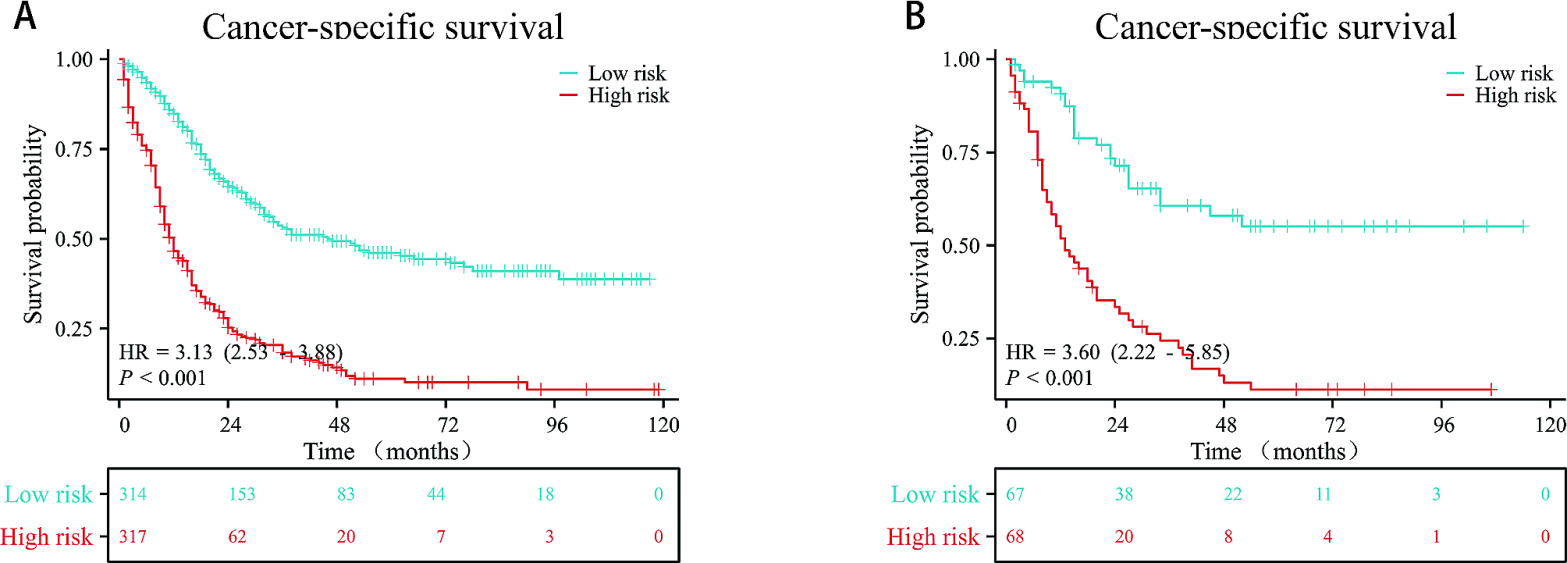
Kaplan-Meier curves of CSS for patients by stratifying individual scores from the nomogram into low and high-risk groups. **A** In the training cohort; **B** In the verification cohort. CSS, cancer-specific survival

We constructed a nomogram model that includes TD, N stage, T stage, TNM stage, CEA, and chemotherapy in the training cohort (Fig. 3A). In this study, the training cohort was used as the internal validation, and the validation cohort was used as the external validation, through internal and external validation, the C-index of the model is 0.721 and 0.713, respectively. The area under the curve (AUC) values for 1-, 3- and 5-year CSS in the internal validation were 0.76, 0.791, and 0.817, respectively (Fig. 3B), while in the external cohort, the corresponding values were 0.791, 0.765, and 0.848 (Fig. 3C). Calibration curves of 3- and 5-year indicated good calibration for both internal validation (Fig. 3D and E) and external validation (Fig. 3F and G). Moreover, when stratifying individual scores from the nomogram into low and high-risk groups and plotting Kaplan-Meier survival curves, significant prognostic differences were observed in both the training cohort (Fig. 4A) and validation cohort (Fig. 4B).

## Discussion

Colorectal SRCC is known for its distinct morphology and dire prognosis. The insidious onset of colorectal SRCC stands out as one of the important factors contributing to its dismal prognosis. A retrospective study involving 37 patients with colorectal SRCC found that 89% of patients presented with advanced-stage disease at the time of diagnosis, with 46% classified as stage III and 43% as stage IV [18]. Constrained by its rarity, current studies on colorectal SRCC mostly rely on small sample sizes. Furthermore, there are presently no markers clinically available to assess the prognosis of colorectal SRCC patients. Given that colorectal SRCC is frequently diagnosed at advanced stages, we conducted a multicenter cohort study to evaluate prognostic markers impacting the outcome of stage III/IV colorectal SRCC.

In CRC patients, the presence of TD without lymph node metastasis is defined as stage N1c [14]. There is controversy in the medical community regarding the prognostic value of TD, as it is believed to be considered only in the absence of lymph node metastasis, potentially underestimating the severity of the disease [19, 20]. Goldstein and Turner reported that TD as an independent adverse prognostic factor in CRC, should be distinguished from lymph node metastasis (LNM). They asserted that TD was more common in advanced tumors, and that TD-positive patients had lower 5-year survival than LNM positive patients [21]. The effect of TD positivity on the clinicopathological characteristics and prognosis of colorectal SRCC is rarely reported.

This study combined the SEER database and a multi-center expanded sample size to investigate the value of TD in the prognosis of colorectal SRCC. We report for the first time that TD positivity is an independent factor strongly related to the poor prognosis of this rare tumor type. In our study, the overall positive rate of TD in stage III/IV colorectal SRCC ranges between 41.5 and 49.8%, which is significantly higher than that observed in stage III/IV CRC (16.3–27.1%) [22, 23]. The 5-year OS of stage III/IV colorectal SRCC in our study was 23.5-27.2%, which was similar to the results of a Korean study [24] and significantly lower than that of stage III/IV CRC (32.3-80.7%) [25]. Further stratified analysis showed that TD positivity was significantly associated with worse CSS and OS in stage III patients, but not in stage IV patients. This may be due to the fact that in stage IV colorectal SRCC, patients often has entered the stage of tumor dissemination and progression. Compared to other types of CRC, colorectal SRCC exhibits higher mutation frequencies of TP53, KIT, and BRAF, and lower frequencies of PIK3CA, KRAS, ATM, and APC mutations [2, 5]. Moreover, SRCC’s colloid-like characteristics hinder the recognition of host immune cells, and reduced expression of E-cadherin and β-catenin leads to loose intercellular junctions [26]. These characteristics may further contribute to colorectal SRCC aggressive behavior.

In terms of clinicopathological features, we found several significant differences between stage III/IV colorectal SRCC and stage III/IV CRC [23]. In our study, he proportion of TD-positive colorectal SRCC cases with a tumor diameter exceeding 5 cm was between 50.0 and 64.0%, whereas this proportion was 38.0-46.1% in stage III/IV CRC. Similarly, the proportions of patients in T4 (64.3-72%) and N2 (76.8-85.7%) stages were significantly higher in colorectal SRCC compared to those with stage III/IV CRC (26.5-49.1% and 30.0-47.7%, respectively). These results suggest that the positive rate of TD is closely associated with tumor size, local invasion, multiple lymph node metastasis. Additionally, a study had identified differences in gene and protein expression between TD and LNM in CRC [27]. Specifically, the proteins SFRP2 and MXRA5 were found to be significantly upregulated in TD. SFRP2 is thought to collaborate with WNT16B to prevent cell death and promote proliferation, migration, and invasion [28], while MXRA5 functions as a matrix remodeling molecule and a cell adhesion molecule. Both SFRP2 and MXRA5 are linked to a poor prognosis [29]. These molecular differences enable TD to exhibit enhanced cell motility, matrix remodeling, and epithelial-to-mesenchymal transition (EMT). These variances also lead to a distinct composition of the tumor microenvironment (TME), characterized by increased levels of fibroblasts, macrophages, and regulatory T cells [27]. These findings reflect the aggressive biological nature of TD and provide insights into the clinicopathological characteristics observed in TD-positive patients.

At present, there is no prognostic model in stage III/IV colorectal SRCC. We further constructed a nomogram model based on TD positivity in the training cohort. Through internal and external validation, the model demonstrated good calibration, and the area under the curve confirmed its accuracy. Within this nomogram model, individual scores were categorized into low-risk and high-risk groups. Subsequently, Kaplan-Meier curves were plotted for different risk groups, and the results showed a significant stratification of patient prognosis, demonstrating good discrimination of this model, and providing an effective prediction tool for clinical management and service of stage III/IV SRCC patients.

This study has some limitations. Firstly, retrospective data analysis may lead to data gaps, potentially affecting the representativeness of the results. we partly addressed this problem by utilizing the SEER database as the training cohort and data from four tertiary medical institutions in China as the validation cohort. Secondly, the prognostic value of TD positivity in stage IV colorectal SRCC is not clearly established, and further analysis is needed to explore possible reasons within this subgroup.

In summary, TD positivity can serve as a prognostic marker for advanced colorectal SRCC, and the nomogram model based on TD positivity can be used as a prognostic prediction tool for advanced colorectal SRCC.

### Electronic supplementary material

Below is the link to the electronic supplementary material.

Supplementary Material 1

## Data Availability

The data that support the findings of this study are available on request from the corresponding author.

## Abbreviations

CRC: Colorectal cancer
SRCC: Colorectal signet-ring cell carcinoma
TD: Tumor deposits
AJCC: American Joint Committee on Cancer
CSS: Cancer-Specific Survival
OS: Overall Survival
CEA: Carcinoembryonic antigen
PNI: Perineural invasion
AUC: Area under the curve
EMT: Epithelial-to-mesenchymal transition
TME: Tumor microenvironment

## References

[1] Brenner H, Chen C (2018). The colorectal cancer epidemic: challenges and opportunities for primary, secondary and tertiary prevention. Br J Cancer.

[2] Shi T, Huang M, Han D (2019). Chemotherapy is associated with increased survival from colorectal signet ring cell carcinoma with distant metastasis: a Surveillance, Epidemiology, and end results database analysis. Cancer Med.

[3] Fadel MG, Malietzis G, Constantinides V (2021). Clinicopathological factors and survival outcomes of signet-ring cell and mucinous carcinoma versus adenocarcinoma of the colon and rectum: a systematic review and meta-analysis. Discov Oncol.

[4] Benesch MGK, Mathieson A. Epidemiology of Signet Ring Cell Adenocarcinomas. Cancers (Basel) 2020; 12. 10.3390/cancers12061544 PMC7352645 32545410

[5] Alvi MA, Loughrey MB, Dunne P (2017). Molecular profiling of signet ring cell colorectal cancer provides a strong rationale for genomic targeted and immune checkpoint inhibitor therapies. Br J Cancer.

[6] Wang R, Ma X, Li Y (2016). The characteristics and Prognostic Effect of E-Cadherin expression in colorectal Signet Ring Cell Carcinoma. PLoS ONE.

[7] Nitsche U, Zimmermann A, Spath C (2013). Mucinous and signet-ring cell colorectal cancers differ from classical adenocarcinomas in tumor biology and prognosis. Ann Surg.

[8] Wong-Chong N, Motl J, Hwang G (2018). Impact of Tumor deposits on oncologic outcomes in stage III Colon cancer. Dis Colon Rectum.

[9] Li J, Yang S, Hu J (2016). Tumor deposits counted as positive lymph nodes in TNM staging for advanced colorectal cancer: a retrospective multicenter study. Oncotarget.

[10] Mirkin KA, Kulaylat AS, Hollenbeak CS, Messaris E (2018). Prognostic significance of Tumor deposits in stage III Colon cancer. Ann Surg Oncol.

[11] Brouwer NPM, Nagtegaal ID (2021). Tumor deposits improve staging in colon cancer: what are the next steps?. Ann Oncol.

[12] Sobin LH, Hermanek P, Hutter RV (1988). TNM classification of malignant tumors. A comparison between the new (1987) and the old editions. Cancer.

[13] Edge SB, Compton CC (2010). The American Joint Committee on Cancer: the 7th edition of the AJCC cancer staging manual and the future of TNM. Ann Surg Oncol.

[14] Amin MB, Greene FL, Edge SB (2017). The Eighth Edition AJCC Cancer staging Manual: continuing to build a bridge from a population-based to a more personalized approach to cancer staging. CA Cancer J Clin.

[15] Cohen R, Shi Q, Meyers J (2021). Combining tumor deposits with the number of lymph node metastases to improve the prognostic accuracy in stage III colon cancer: a post hoc analysis of the CALGB/SWOG 80702 phase III study (Alliance)(☆). Ann Oncol.

[16] Konishi T, Shimada Y, Hsu M (2018). Association of Preoperative and Postoperative Serum Carcinoembryonic Antigen and Colon cancer outcome. JAMA Oncol.

[17] Imperial R, Ahmed Z, Toor OM (2018). Comparative proteogenomic analysis of right-sided colon cancer, left-sided colon cancer and rectal cancer reveals distinct mutational profiles. Mol Cancer.

[18] Liang Z, Yan D, Li G, Cheng H (2018). Clinical analysis of primary colorectal Signet-Ring Cell Carcinoma. Clin Colorectal Cancer.

[19] Delattre JF, Selcen Oguz Erdogan A, Cohen R (2022). A comprehensive overview of tumour deposits in colorectal cancer: towards a next TNM classification. Cancer Treat Rev.

[20] Ueno H, Nagtegaal ID, Quirke P (2023). Tumor deposits in colorectal cancer: Refining their definition in the TNM system. Ann Gastroenterol Surg.

[21] Goldstein NS, Turner JR (2000). Pericolonic tumor deposits in patients with T3N + MO colon adenocarcinomas: markers of reduced disease free survival and intra-abdominal metastases and their implications for TNM classification. Cancer.

[22] Wu WX, Zhang DK, Chen SX (2022). Prognostic impact of tumor deposits on overall survival in colorectal cancer: based on Surveillance, Epidemiology, and end results database. World J Gastrointest Oncol.

[23] Liu C, Tian M, Pei H (2022). Prognostic value of the N1c in Stage III and IV Colorectal Cancer: a propensity score matching study based on the Surveillance, Epidemiology, and end results (SEER) database. J Invest Surg.

[24] Kim JH, Kim H, Kim JW, Kim HM. Trends in the incidence and Survival Rates of Colorectal Signet-Ring Cell Carcinoma in the South Korean Population: analysis of the Korea Central Cancer Registry Database. J Clin Med 2021; 10. 10.3390/jcm10184258 PMC8468145 34575368

[25] Cardoso R, Guo F, Heisser T (2022). Overall and stage-specific survival of patients with screen-detected colorectal cancer in European countries: a population-based study in 9 countries. Lancet Reg Health Eur.

[26] Borger ME, Gosens MJ, Jeuken JW (2007). Signet ring cell differentiation in mucinous colorectal carcinoma. J Pathol.

[27] Brouwer NP, Webbink L, Haddad TS (2023). Transcriptomics and proteomics reveal distinct biology for lymph node metastases and tumour deposits in colorectal cancer. J Pathol.

[28] Sun Y, Zhu D, Chen F (2016). SFRP2 augments WNT16B signaling to promote therapeutic resistance in the damaged tumor microenvironment. Oncogene.

[29] Wang GH, Yao L, Xu HW (2013). Identification of MXRA5 as a novel biomarker in colorectal cancer. Oncol Lett.

