# Supplementary figures and images for: Prognostic and predictive value of tumor deposits in advanced signet ring cell colorectal cancer: SEER database analysis and multicenter validation
Source: World J Surg Oncol. 2024 Apr 22;22:107. doi: 10.1186/s12957-024-03362-0 (PMC11034099; doi:10.1186/s12957-024-03362-0)

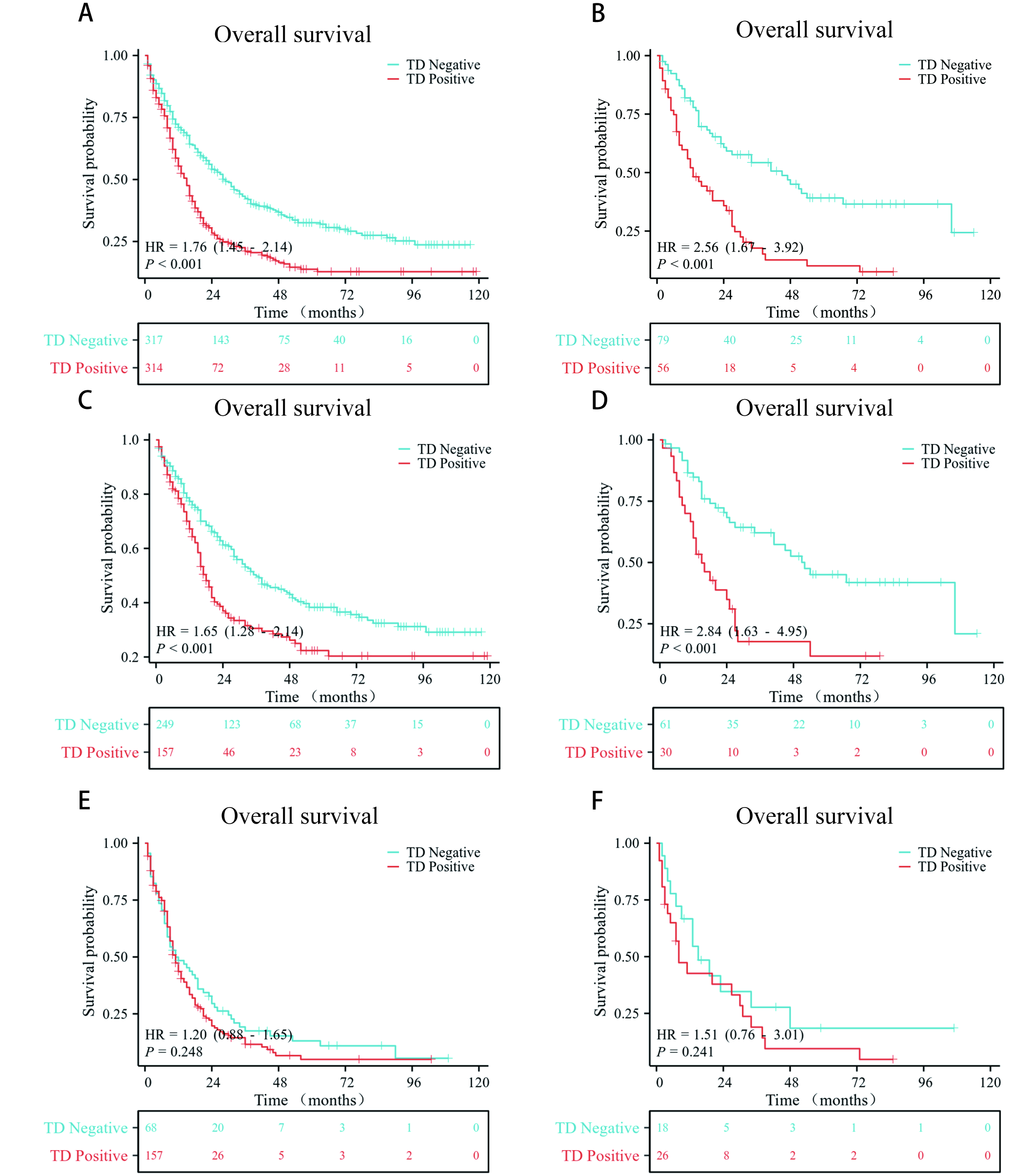

Supplement: Supplementary file 1 — Supplementary Material 1 [file 12957_2024_3362_MOESM1_ESM.tif]
